# Analysis of ionizing radiation doses received by patients during electrocardiological procedures

**DOI:** 10.1007/s11845-024-03717-2

**Published:** 2024-05-22

**Authors:** Michał Biegała, Łukasz Ząbczyński, Maria Anna Staniszewska

**Affiliations:** 1 Department of Medical Imaging Technology, Faculty of Medicine, Medical University of Lodz, Lindleya 6, 90-131 Lodz, Poland; 2 TMS Sp. z o.o., Warsaw, Poland; 3 Department of Medical Physics, Copernicus Memorial Hospital in Lodz Comprehensive Cancer Center and Traumatology, Lodz, Poland

**Keywords:** Cardioverter-defibrillators, DAP, Electrocardiological procedures, Exposure to ionizing radiation, Pacemaker, RF ablation

## Abstract

Medical procedures in the field of electrocardiology belong to a large group of cardiological procedures. Performing them involves exposure to ionizing radiation. In this study, five medical procedures in the field of electrocardiology performed in three medical facilities were analyzed in terms of patients’ exposure to ionizing radiation. A total of 178 patients were analyzed. Depending on the type of procedure, the recorded doses ranged from 7.4 to 614.62 mGy. The majority of electrocardiological procedures are pacemaker implantations 38% and RF ablation 33%. The results obtained show a significant dispersion of the recorded dose values in the same type of treatment. This is reflected, for example, in the high coefficient of variation for doses in RF ablation. The type of X-ray machine used during the procedure also influences the dose values. Although the exposure of patients undergoing electrocardiological procedures to ionizing radiation is much lower than in the case of cardiac vascular procedures, it may reach a similar level, especially in the case of implantation of devices regulating the correct functioning of the heart.

## Introduction

Electrocardiological procedures belong to a large group of interventional cardiology procedures. Unlike vascular therapy procedures, they generally do not result in excessive doses to patients undergoing them, although, of course, unavoidable exposure to X-rays should not be ignored [1–6]. Electrocardiological procedures do not require the use of technologically advanced X-ray equipment that is required for vascular therapy procedures: they can be performed under the control of a C arm device (with the option of pulsed fluoroscopy).

However, as in the case of other interventional cardiology procedures, cardiologists and cardiac surgeons are authorized to perform electrocardiological procedures. Electrocardiological procedures essentially involve electrophysiological intervention, which is based on the use of devices that regulate and support the heart. These devices include the following: pacemakers, cardiac resynchronization therapy (CRT) devices, and implantable cardioverter defibrillators (ICD). Electrophysiological procedures also include radio frequency electromagnetic field ablation (RF ablations) and cryoablation [7].

Pacemakers are implanted when there is a need to stimulate the heart in the event of automaticity or conduction disorders [8]. The purpose of the pacemaker is to monitor the heart’s activity and generate an electrical impulse when an abnormality is detected. A pacemaker may be used if the heart’s natural pacemaker, the sinoatrial node, is not working properly and causes the heart rate to slow down.

A special type of pacemaker is the double-chamber pacemaker — CRT. It is used if the ventricles do not contract at the same time, causing heart failure to worsen. The task of CRT is to synchronize the stimulation of both ventricles. The right ventricle is stimulated by an electrode intended for cardioversion/defibrillation. This electrode is placed in the apex of the right ventricle. The left ventricle is stimulated by an electrode that is inserted through the coronary sinus into the epicardial vein, which is located on the lateral or posterior wall of the left ventricle. An additional CRT electrode is placed in the right atrium of the heart.

Resynchronization therapy devices are implanted for stimulation that corrects electrical and mechanical desynchrony of the heart in patients with heart failure and a wide QRS complex optimally treated pharmacologically [9].

A device similar in structure to a pacemaker is a cardioverter-defibrillator (ICD), which can generate a high-energy impulse used to stop abnormal heart rhythms and restore normal ones.

Cardioverter defibrillators (ICDs) are implanted when it is necessary to detect and recognize life-threatening arrhythmias, interrupt arrhythmias, and prevent sudden cardiac death [10]. All the abovementioned devices require the insertion of wires and electrodes into appropriate locations in the heart space, which is done under the control of X-ray radiation. The aim of this study is to analyze the doses received by patients undergoing routine electrocardiological procedures.

Ablations are a separate cardiac procedure. These are minimally invasive procedures aimed at destroying or isolating the area of heart tissue that is responsible for the occurrence of arrhythmia. The procedure involves creating a small scar that prevents the conduction of impulses that induce cardiac arrhythmias. This involves the use of ablation electrodes which are inserted into the heart. Ablation is performed using a radiofrequency (RF) electromagnetic field causing a thermal effect (so-called RF ablation) or by strongly cooling the tissue (so-called cryoablation), where the cooling agent is nitrous oxide. As a result of ablation, small, homogeneous, and necrotic changes occur at the point of contact of the electrode with the heart tissues. In this way, the area of the heart that is responsible for the development of abnormal heart rhythms is ultimately damaged.

## Material and methods

The measurements were performed in three centers with the highest level of reference where procedures in the field of interventional cardiology are performed. These procedures were performed by specialist physicians with at least 5 years of experience in performing this type of procedure.

Data from 178 adult patients undergoing the following procedures were analyzed:

- Implantation of single-chamber and double-chamber pacemakers
- Implantation of cardioverter-defibrillators
- Implantation of resynchronization pacemakers
- RF ablation
- Cryoablation

Patient data collected included the following: type of procedure, input kerma value at the reference point (referred to as “dose”), duration of fluoroscopy during the procedure, patient gender, and patient weight (recorded only in two centers). The above data were obtained from patient records provided by cardiology centers.

The values of input kerma value at the reference point (referred to as “dose”) were displayed (and then recorded) by the radiological devices after finishing of the procedure the patients. In two facilities, a Ziehm C-arm (Germany, Norymberga) device was used for procedures, and in the third, an Infinix series angiograph from Canon (Japan, Tokyo) was used for this purpose.

The statistical analysis of the results obtained in this work was performed using Statistica 13.1 software. The Pearson *R* correlation coefficient and its statistical significance were determined. The significance level adopted was *α* = 0.05; therefore, *p* < 0.05 confirms the statistical significance.

## Results

The data collected in this work was systematized according to types of procedures. Using the Statistica 13.1 software, the average dose values were calculated along with the standard deviation, and the minimum and maximum values of these doses were indicated.

The results are presented in Table 1. Table 1 indicates the center from which the data come from but does not include the division by patient gender in order to maintain group sizes that guarantee representative averages — especially in less frequently performed procedures. Moreover, after analyzing the source data, it was found that the dose values are in no way determined by the patient’s gender.

**Table 1:** Doses for specific types of electrocardiological procedures

Type of procedure	Number of patients	Center number	Dose (mGy)		
			Mean dose ± standard deviation	Minimum	Maximum
Pacemakers	37	1	74.09 ± 44.60	20.73	189.30
	32	3	28.16 ± 33.13	4.00	179.00
ICD	12	1	64.08 ± 47.96	22.45	200.88
	5	3	49.70 ± 27.37	14.98	403.00
CRT	10	1	308.50 ± 124.50	127.34	614.62
	10	3	94.40 ± 79.60	18.30	286.00
RF ablation	16	2	179.72 ± 175.66	16.60	608.97
	45	3	70.80 ± 65.50	7.40	310.47
KRIO	11	3	93.70 ± 42.00	45.00	180.00

**Fig. 1:**
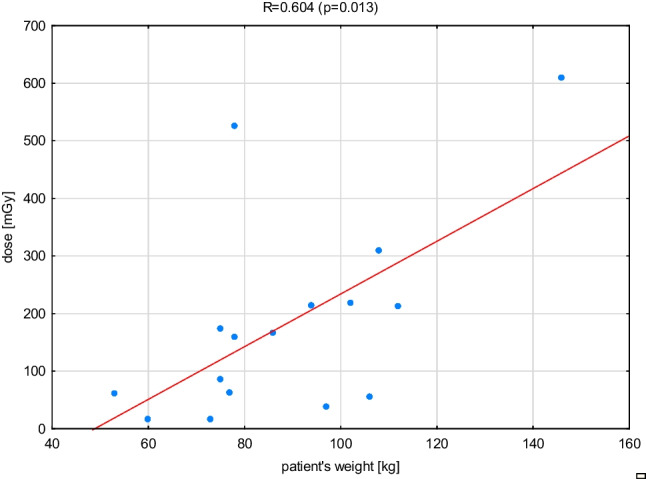
The relationship between dose and patient’s weight during RF ablation

**Fig. 2:**
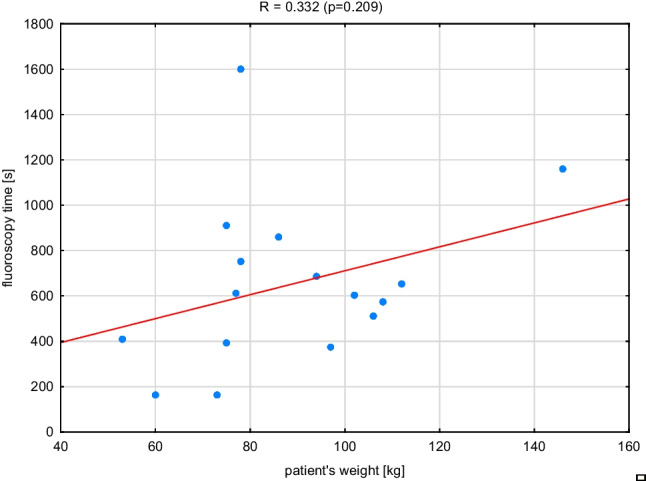
The relationship between fluoroscopy time and patient’s weight in RF ablation procedure

**Fig. 3:**
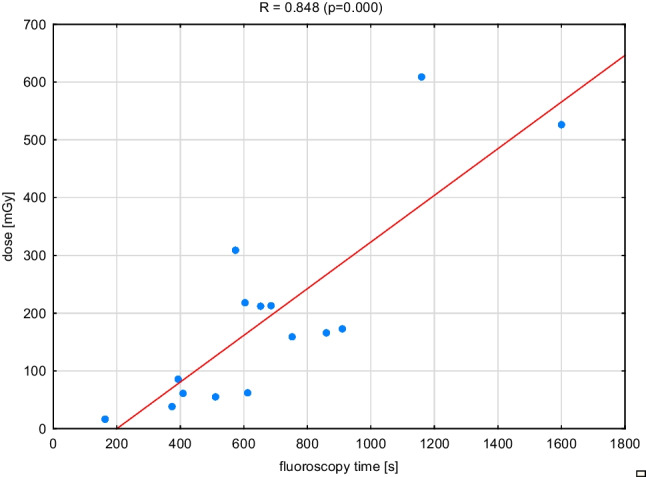
The relationship between the dose received by the patient and the fluoroscopy time during RF ablation

In order to check the relationship between the dose received by the patient, his weight, and fluoroscopy time, the results of RF ablation performed in center 2 were analyzed (data from this center concern only this procedure). The results are shown in Figs. 1, 2, and 3, where the Pearson *R* coefficient and the statistical significance of this coefficient are given.

## Discussion

Data collected in the three analyzed cardiology centers, presented in Table 1, indicate that the majority of electrocardiological procedures are pacemaker implantations (approx. 38% of procedures) and RF ablation (approx. 33% of procedures). Cryoablation was performed much less frequently and only in one of the three analyzed centers. Cardiac pacemakers (ICD, CRT) were also implanted much less frequently — approximately 10% of each type of procedure.

Also noteworthy is the significant dispersion of recorded dose values in the same type of treatment, reaching two orders of magnitude. This is reflected, for example, in the high coefficient of variation for RF ablation doses performed at center 2, which reaches 100% (the coefficient of variation C.V. is the quotient of the standard deviation and the mean, expressed as a percentage). This most likely indicates a very individual course of these procedures, strongly dependent on the clinical and anatomical conditions of the patient — especially since it is visible in all three centers. The type of X-ray machine used during the procedure also influences the dose values: the average dose values in center 3, where an angiograph was used, are up to three times lower than the dose values in the same procedures in centers 1 and 2, where a C-arm machine was used.

The results illustrated in Figs. 1, 2, and 3 prove that the dose-to-patient weight relationship is characterized by a moderate correlation *R* = 0.604 (*p* = 0.013) — although statistically significant, while the relationship between fluoroscopy time and patient weight is characterized by a weak correlation *R* = 0.332 (*p* = 0.209). Only the dose and fluoroscopy time dependence are strongly correlated *R* = 0.848 (*p* = 0.000) — with a statistically significant correlation. The presented relationships show that the patient’s weight is not an important factor in determining his exposure level in electrocardiological procedures.

The dose values recorded as part of this study were compared with adequate data from the literature, which are not very numerous and have a very different level of detail. It should be emphasized that although in the case of interventional radiology procedures under the control of X-ray machines, the kerma in the air at the reference point is the most representative value for assessing the patient’s exposure, most published works provide the values of the product of the dose (or kerma) and the primary surface area X-ray beam (DAP/KAP) and possibly the effective dose without information about the algorithm for its calculation.

In accordance with Polish legal regulations [11], the kerma value at the reference point is obligatorily recorded for patients undergoing interventional radiology procedures, and its level determines any medical actions taken in relation to the patient. Therefore, it was only possible to compare the results of this study with a few studies in which the size of the kerma in the air at the reference point was operated. This comparison is presented in Table 2.

**Table 2:** Comparison of dose values for patients in electrocardiological procedures obtained in this study with adequate data from the literature

Type of procedure	Range of dose values (i.e. kerma at the reference point) [mGy]	Data source
Pacemakers	20–150	[12]
	4–189	This publication
ICD	20–150	[12]
	15–403	This publication
SH (*)	800–1208	[13]

SH(*), according to [13] “interventions to treat structural heart (SH) disease, (…) PCI is excluded”, i.e., therapeutic activities in structural heart diseases, not including PCI

The dose values in Table 2 show a significant range of values, which is probably the result of differences in equipment, operator skill, and, most importantly, differences in the clinical condition and anatomy of patients.

It should be taken into account that the implantation of various types of cardiostimulation devices is being considered, which involves the need to introduce a different number of electrodes to different places inside the heart. Therefore, the anatomical and clinical conditions of individual patients are very important, which results in a significant variation in the doses they receive.

The characteristics of the X-ray machines used during procedures and specifically their dose calibration (set by the manufacturer) are also important. Significantly lower doses will be recorded during procedures performed under angiograph control than during procedures performed under C-arm control (although such devices are completely sufficient for performing electrocardiological procedures).

Interventional cardiology procedures performed much more often than electrocardiological procedures are procedures involving coronary vessels, during which the doses received by patients are much higher — even by an order of magnitude, as illustrated by the data presented in Table 3.

**Table 3:** Doses received by patients during vascular interventional cardiology procedures (based on data from the literature)

Type of procedure	Range of dose values (i.e., kerma at the reference point) (mGy)	Data source
Coronary angiography	260–670	[12]
	451–712	[13]
	320	[14]
PCI	510–2750	[12]
	1123–1900	[13]
	790	[14]
	16–571 (*)	[15]

(*) data refer to children aged 4 months to 18 years

## Conclusions

The results presented in the work allow for drawing the following conclusions:

- Although the exposure of patients undergoing electrocardiological procedures is much lower than in the case of cardiac vascular procedures, it may reach a similar level — especially in implantation procedures of devices regulating the correct functioning of the heart
- The level of doses received by patients depends on the type of radiological device used to monitor the course of the procedure
- A significant range of dose values obtained during the same type of procedure probably results from differences in the clinical condition and anatomical structure of patients.

## Data Availability

The data supporting the conclusions of this study and used in this study are not publicly available for sensitivity reasons and are available from the corresponding author upon reasonable request. The data is located in a controlled-access data storage at the Medical University of Łódź.
